# Lipid A-Mediated Bacterial–Host Chemical Ecology: Synthetic Research of Bacterial Lipid As and Their Development as Adjuvants

**DOI:** 10.3390/molecules26206294

**Published:** 2021-10-18

**Authors:** Atsushi Shimoyama, Koichi Fukase

**Affiliations:** 1 Department of Chemistry, Graduate School of Science, Osaka University, 1-1 Machikaneyama, Toyonaka 560-0043, Osaka, Japan; 2 Project Research Center for Fundamental Sciences, Osaka University, 1-1 Machikaneyama, Toyonaka 560-0043, Osaka, Japan

**Keywords:** lipopolysaccharide, lipid A, glycolipid, natural product chemistry, chemical biology, chemical ecology, adjuvant, symbiotic bacteria, *Alcaligenes faecalis*

## Abstract

Gram-negative bacterial cell surface component lipopolysaccharide (LPS) and its active principle, lipid A, exhibit immunostimulatory effects and have the potential to act as adjuvants. However, canonical LPS acts as an endotoxin by hyperstimulating the immune response. Therefore, LPS and lipid A must be structurally modified to minimize their toxic effects while maintaining their adjuvant effect for application as vaccine adjuvants. In the field of chemical ecology research, various biological phenomena occurring among organisms are considered molecular interactions. Recently, the hypothesis has been proposed that LPS and lipid A mediate bacterial–host chemical ecology to regulate various host biological phenomena, mainly immunity. Parasitic and symbiotic bacteria inhabiting the host are predicted to possess low-toxicity immunomodulators due to the chemical structural changes of their LPS caused by co-evolution with the host. Studies on the chemical synthesis and functional evaluation of their lipid As have been developed to test this hypothesis and to apply them to low-toxicity and safe adjuvants.

## 1. Introduction

The immunomodulatory effects triggered by bacteria have long been identified [1]. Tumor burden decrease and regression caused by bacterial infections have been reported for over 300 years [2]. In 1893, Coley was the first to try cancer immunotherapy using *Streptococcus pyogenes* and *Serratia marcescens* [3]. The immunostimulatory effects of killed *Salmonella typhimurium* and *Mycobacterium tuberculosis* were confirmed in 1916 and 1924, respectively. These immunostimulatory effects are now widely known as the innate immune system. Innate immunity is stimulated via various innate immune receptors in multicellular organisms by recognizing the molecular pattern of pathogens. Since innate immune responses promote acquired immune responses including antigen–antibody reactions, the development of innate immune stimulators as adjuvants, vaccine ingredients that promote antibody production, have been actively investigated. On the other hand, most bacteria-derived innate immune stimulators exhibit inflammatory effects and toxicity. For example, *Escherichia coli* cell surface component lipopolysaccharide (LPS) can cause a cytokine storm, leading to lethal sepsis [1]. Therefore, regulating and attenuating the toxicity of innate immune stimulators is essential for their application as adjuvants.

**Figure 1:**
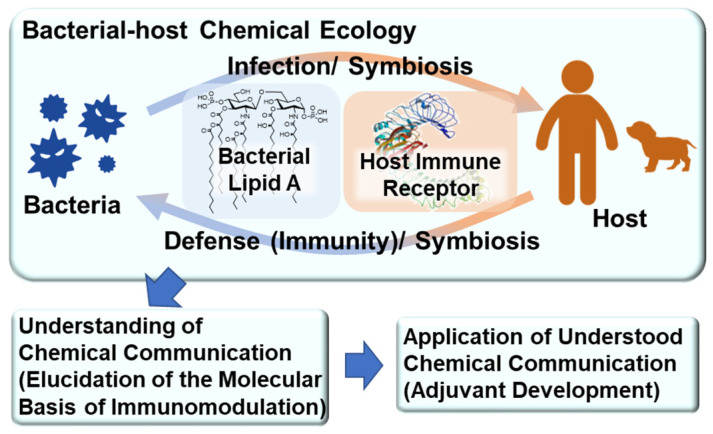
Bacterial–host chemical ecology mediated by lipid A.

The field of chemical ecology research considers various biological phenomena among living organisms as molecular interactions (chemical communication). We have revealed the relationship between immune function and the chemical structure of Gram-negative bacterial cell surface components, such as LPS and its active principle glycolipid lipid A. We recently hypothesized that LPS and lipid A mediate bacterial–host chemical ecology to regulate various biological phenomena in the host, mainly immunity (Figure 1). We predicted that parasitic and symbiotic bacteria inhabiting the host possess low-toxicity immunomodulators due to the chemical structural changes of their LPS caused by co-evolution with the host. Thus, we synthesized parasitic and symbiotic bacterial lipid As, evaluated their immune functions, and developed research to utilize them as low-toxicity and safe vaccine adjuvants [4,5,6,7].

## 2. Bacterial LPS and Its Active Principle Lipid A, an Innate Immune Stimulator

LPS, a major glycoconjugate in the outer membrane of Gram-negative bacteria, is a well-known innate immune stimulator. First, we would like to introduce the history of LPS as an immune stimulatory molecule.

**Figure 2:**
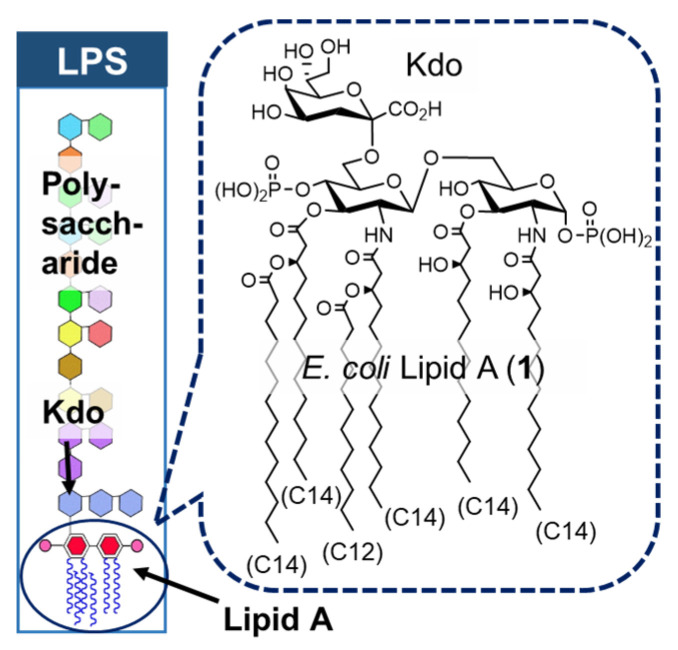
*E. coli* LPS and Kdo-lipid A.

In 1892, Pfeiffer (a pupil of the bacteriologist Koch) showed that *Vibrio cholerae* has two distinct toxic components: a heat-sensitive exotoxin and a heat-stable endotoxin [8]. In 1945, Westphal reported that LPS, a component of the outer membrane of Gram-negative bacteria, was the active ingredient of endotoxins [8]. In 1957, glycolipid lipid A, an acylated disaccharide at the terminus of LPS, was reported to be the active principle of LPS [8]. Shiba and Kusumoto, in collaboration with a German research group, submitted the corrected structure of *E. coli* lipid A (**1**) (Figure 2), and in 1985, they achieved the first chemical synthesis of *E. coli* lipid A (**1**) to confirm that lipid A is the active principle of endotoxin [9,10,11]. At the same time, Qureshi and Takayama also identified the lipid A structure [12]. It has also been shown that lipid A is bound to the terminal of the polysaccharide portion of LPS via the specific acidic sugar Kdo (2-keto-3-deoxy-d-mannooctanoic acid) (Figure 2).

**Figure 3:**
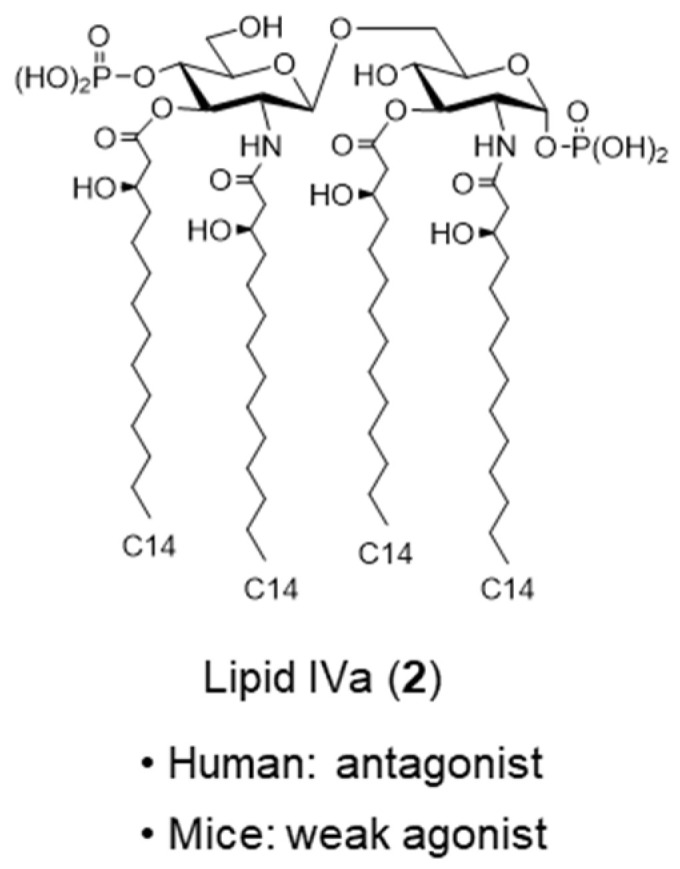
Lipid IVa.

Shiba and Kusumoto also successfully synthesized lipid IVa (**2**) (Figure 3), a precursor of *E. coli* lipid A (**1**), and found that lipid IVa (**2**) acts as an immunostimulator in mice but acts as an antagonist in humans [13,14]. The discovery of antagonists implied the existence of receptors, which prompted further research on the identification of LPS receptors. In 1996, Hoffman found that the Toll gene, which regulates the formation of the Drosophila dorsoventral axis and is essential for defense systems against fungi, leading to a breakthrough that opened the path to the discovery of various innate immune receptors [15]. In 1997, Toll-like receptors (TLRs) were identified as human homologs of the Toll protein in Drosophila [16], and in 1998, Beutler identified TLR4 as the LPS receptor [17]. To date, 10 types of TLRs have been identified in humans (TLR1–10) and 12 types in mice (TLR1–9 and TLR11–13).

**Figure 4:**
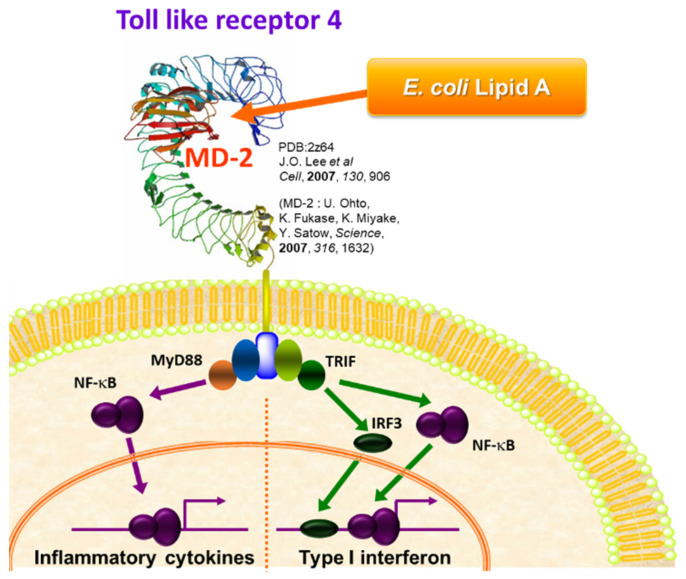
Innate immune activation via TLR4/MD2.

TLRs are membrane glycoproteins with a leucine-rich repeat motif in the ectodomain and a cytoplasmic signaling domain homologous to the interleukin-1 receptor (IL-1R) called the Toll/IL-1R (TIR) domain. TLR4 signals are transduced through various adapter molecules (MyD88, TRIF, TIRAP, and TRAM) (Figure 4) [18]. MyD88-dependent signaling triggers the activation of NF-κB, a transcription factor associated with inflammation, which produces proinflammatory cytokines such as tumor necrosis factor (TNF)-α and IL-6, which in turn induces a defense response against infection. On the other hand, TRIF-dependent signaling activates interferon (IFN) regulator 3 (IRF3), which triggers the production of antiviral cytokine type I IFN. It has also been shown that the canonical *E. coli* LPS strongly activates both signals simultaneously (Figure 4), causing a severe inflammatory response that results in lethal toxicity. Therefore, it is essential to attenuate the function of lipid A by regulating the TLR4 signaling pathway for the development of lipid A-based adjuvants.

**Figure 5:**
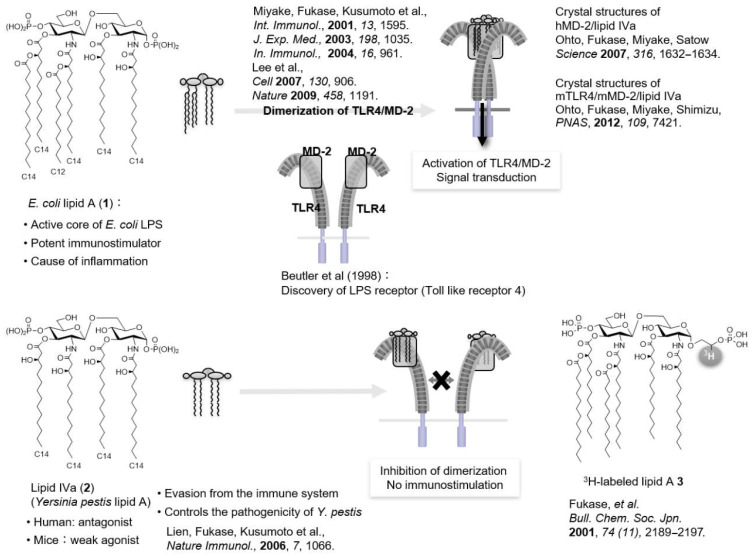
Molecular mechanism of TLR4/MD2 dimerization process.

**Figure 6:**
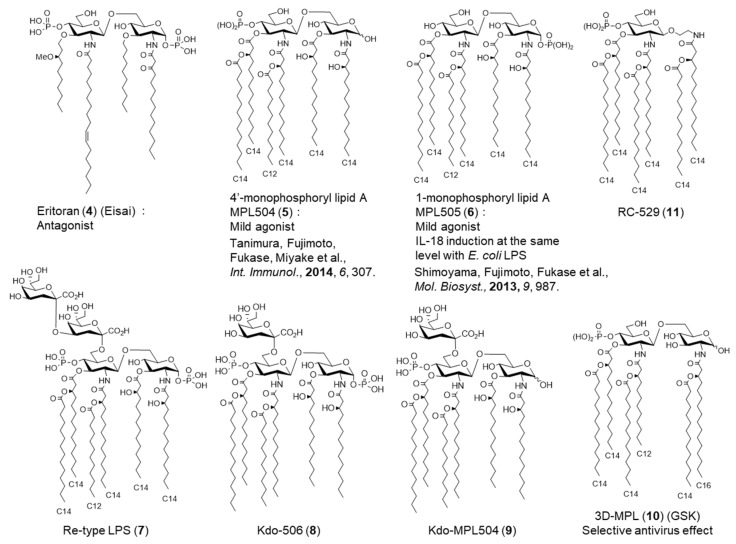
Chemical structures of various lipid As and lipid A analogs.

It is essential to elucidate the molecular basis of lipid A recognition by TLR4 to regulate TLR4 signaling and attenuate the function of lipid A. Miyake found that MD2, an accessory protein, is essential for TLR4 signaling [19]. We synthesized radiolabeled *E. coli* lipid A analog **3** (Figure 5) and investigated the interaction of lipid A with the TLR4/MD-2 complex in collaboration with Miyake [20]. Miyake also revealed that the observed species specificity of TLR4/MD-2 is caused by differences in the recognition of lipid A by MD-2 [21]. Furthermore, X-ray crystallography studies revealed the differences in the binding modes of TLR4/MD-2 to agonists and antagonists. In 2007, Ohto and Sato reported the crystal structure of the human MD-2 complex with lipid IVa (**2**) (antagonist) [22], and Lee showed the crystal structure of the mouse TLR4/MD-2 complex with Eritoran (**4**) (Figure 6), a TLR4 antagonist developed by Eisai [23]. In 2009, Lee reported the X-ray crystallography of human TLR4/MD-2 in complex with *E. coli* LPS [24]. It was found that five of the six acyl chains of *E. coli* lipid A (**1**) were placed inside the hydrophobic pocket of MD-2, while the remaining acyl chain interacted with the hydrophobic surface of the adjacent TLR4, and these interactions induced the dimerization of the TLR4/MD-2 complex, activating the innate immune response. The antagonist lipid IVa (**2**) binds to MD-2 in a 180° rotation of lipid A relative to the agonist *E. coli* lipid A (**1**) and does not cause TLR4/MD-2 dimerization, and all acyl chains are accommodated in the pocket of MD-2. Ohto uncovered the crystal structure of the mouse TLR4/MD-2 complex with lipid IVa (**2**). Incidentally, as mentioned above, lipid IVa (**2**) acts as an antagonist in humans but as an agonist in mice [25]. In the case of mice, three of the four acyl chains of lipid IVa (**2**) were housed inside the pocket of MD-2, and the remaining one interacted with the hydrophobic surface of the adjacent TLR4, resulting in TLR4/MD-2 dimerization. Thus, the difference in the binding mode of lipid A to MD-2 was found to have a significant impact on TLR4-mediated immune regulation.

It has been shown that the agonist and antagonist effects of lipid A can be controlled by the number and length of acyl chains and the charge of the phosphate group in previous structure–activity relationship studies (Figure 5 and Figure 6) [26,27,28]. For example, hexa-acylated *E. coli* lipid A (**1**) is an agonist, whereas tetra-acylated lipid IVa (**2**) is an antagonist. MPL504 (**5**) (Figure 6), which is *E. coli* lipid A (**1**) lacking 1-phosphate, showed weaker IL-6 inducing activity than *E. coli* lipid A (**1**) [5,29,30]. Incidentally, the rate of TLR4/MD2 dimerization in response to MPL was much lower than that in response to *E. coli* LPS. Furthermore, compared with *E. coli* lipid A (**1**), MPL504 (**5**) is less dependent on CD14, a glycosylphosphatidylinositol-anchored receptor known to serve as a co-receptor for TLR4. MPL504 (**5**) showed CD14-independent but MyD88-dependent TNFα-induction and TRIF-dependent CD86 upregulation, and IFNβ induction [30]. Like MPL504 (**5**), MPL505 (**6**), which is *E. coli* lipid A (**1**) lacking 4′-phosphate, also exhibits mild immunomodulatory effects. However, MPL504 (**5**) induced IL-18 to a lower extent than *E. coli* LPS, whereas MPL505 (**6**) showed comparable IL-18 induction to *E. coli* LPS [5]. These MPLs are expected to be developed as adjuvants because of their mild immune-activating effects.

LPS is composed of an O-antigen polysaccharide moiety with a characteristic chemical structure for each bacterial species, a core oligosaccharide moiety with high similarity in chemical structure among bacterial species, and a terminal glycolipid lipid A moiety (Figure 2). We also investigated the effect of the core oligosaccharide moiety on lipid A activity. There are R-mutant bacteria that consist of LPS lacking the O-antigen, and the *E. coli* Re-mutant has Re-LPS (**7**) consisting of lipid A attached to the Kdo disaccharide. To evaluate the effect of Kdo on the activity of lipid A, Re-LPS (**7**), Kdo-506 (**8**), and Kdo-MPL504 (**9**) were synthesized (Figure 6), and it was shown for the first time that Kdo enhances the lipid A activity [31].

Thus, it has been suggested that modulating the selectivity and potency of TLR4-MD2 activation could be achieved by structural modification of lipid A.

## 3. Synthetic and Semi-Synthetic Lipid A Adjuvants in Practical Use

GlaxoSmithKline succeeded in attenuating lipid A by optimizing its chemical structure, especially the acyl and phosphate groups, and developed 3D-MPL (**10**) [32] (Figure 6), which has a 4′-monophosphate structure similar to MPL504 (**5**). Currently, it is produced by the derivatization of *Salmonella minnesota* R595 LPS. 3D-MPL (**10**) selectively activates the TRIF-dependent pathway, one of the two signaling pathways downstream of TLR4/MD2, and induces antiviral effects (Figure 4).

GSK developed Adjuvant System (AS) 01, a liposome adjuvant, which is a mixture of 3D-MPL (**10**), cholesterol, and QS21 (a saponin derived from *Kiraja saponaria*, a tree native to South America). AS01 has been applied to the herpes zoster vaccine HZ/su, composed of recombinant glycoprotein E of the varicella zoster virus. When infected with *Plasmodium falciparum*, which causes malaria, infectious sporozoites are injected into human blood via salivary glands during blood collection by *Anopheles* vector mosquitoes. Therefore, vaccines targeting sporozoite surface proteins have been developed. The recombinant protein RTS,S, is composed of a segment of sporozoite protein and a surface antigen of hepatitis B virus, and RTS,S/AS01, a malaria vaccine candidate, has been developed by GSK and is currently in phase III clinical trials. Adjuvant AS02, consisting of 3D-MPL (**10**), oil emulsion, squalene, and QS21 was also developed by GSK. The malaria vaccine RTS,S/AS02, is also in Phase III clinical trials. GSK has also developed adjuvant AS04, a mixture of 3D-MPL (**10**) and aluminum salts. In practice, AS04 is used as an adjuvant for the human papillomavirus (HPV) vaccine Cervarix and the HBV vaccine Fendrix. Furthermore, the MPL mimic RC-529 (**11**) (Figure 6) was approved as an adjuvant in Argentina for the hepatitis B virus vaccine.

Lipid A adjuvants, such as MPL, can induce anti-inflammatory cytokines, such as IL-10, while modulating the induction of inflammatory cytokines, such as IL-6 [33]. Therefore, lipid A adjuvants would have a low risk of developing adjuvant-induced inflammatory diseases, such as autoimmune diseases.

## 4. Lipid A Adjuvants Development Based on Bacterial–Host Chemical Ecology

### 4.1. Parasitic Bacterial Lipid A

In recent years, it has been revealed that some bacteria can stealthily escape the host’s innate immune system. Previously, interesting reports have implied that *Yersinia pestis* evades the host’s innate immunity by modifying its lipid A chemical structure [34,35]. Plague, caused by *Y. pestis*, is an infectious disease in rodents, but it is also transmitted to humans, mainly by fleas. *Y. pestis* produces exotoxin, a proteinaceous toxin that destroys peripheral blood vessels and causes edema and necrosis. Although *Y. pestis* contains various lipid A molecules, including a hexa-acylated form similar to agonistic *E. coli* lipid A (**1**) when incubated at 27 °C, only the antagonistic lipid IVa (**2**) (Figure 3), was produced when incubated at 37 °C, the body temperature of mammals. It was suggested that these differences in the degree of the acylation of lipid A depending on the incubation temperature significantly affect the virulence of *Y. pestis*.

Research on *Y. pestis* lipid A inspired us to study the lipid A-mediated bacterial–host chemical ecology (chemical communication between bacteria and host via lipid A). We hypothesized that the functional analysis of parasitic and symbiotic bacterial lipid A, in which the chemical structure of LPS is modified through co-evolution with the host, would enable the elucidation of the molecular basis for establishing parasitic and symbiotic relationships with the host. Furthermore, we considered that the parasitic and symbiotic bacterial lipid A is assumed to be a low-toxicity component for the establishment of symbiotic relationships and could be a pool of safe immunoregulatory factors that would be promising candidates for vaccine adjuvants. Thus, we developed a chemical biology-based study of parasitic and symbiotic bacterial lipid As.

**Figure 7:**
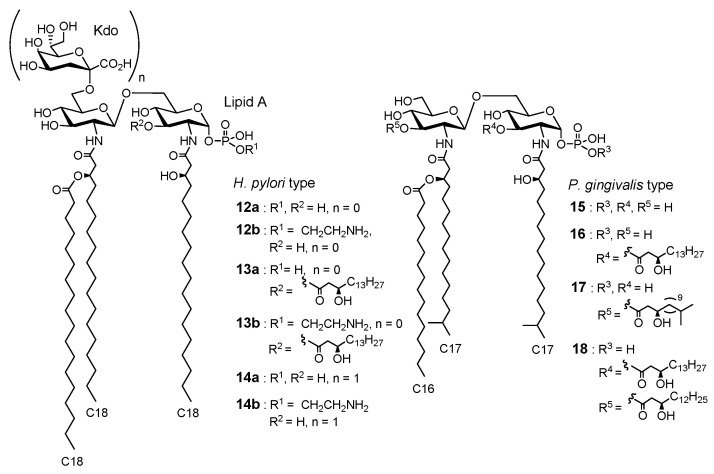
Chemical structures of parasitic bacterial lipid As.

*Helicobacter pylori*, which lives in the stomach, causes gastric ulcers, and *Porphinomonas gingivalis*, an oral bacterium, is a causative agent of periodontal disease. LPS extracted from these parasitic bacteria has weak immunostimulatory effects and has been implicated to be associated with chronic inflammation and atherosclerosis [36,37,38,39]. As described in Figure 7, *H. pylori* lipid As **12**–**14** and *P. gingivalis* lipid As **15**–**18** are characteristic and heterogeneous with several different structures. The selective activation or inhibition of TLR4/MD2 due to the characteristic structure of parasitic bacterial lipid As might be responsible for the aforementioned unique biological activity of these parasitic bacterial LPS [40]. To validate this hypothesis, we synthesized and evaluated parasitic bacterial lipid As **12**–**18**.

Canonical *E. coli* lipid A (**1**) (Figure 2) consists of six fatty chains (C12–C14), whereas *H. pylori* lipid A **12**, **13** has fewer (three to four) but longer (C16–C18) fatty chains. As for the phosphate groups, the canonical *E. coli* lipid A (**1**) has two phosphate groups at the 1 and 4′-positions, whereas *H. pylori* lipid As have only 1-phosphate, that is, *H. pylori* has MPL-type lipid A structures. Lipid A **12a** and **13a** have a normal phosphate group only at the 1-position, while lipid A **12b** and **13b** have an ethanolamine phosphate group only at the 1-position. *P. gingivalis* lipid As **15**–**18** have three to five fatty chains (C15–C17), including chains with terminal branches, and only the 1-position is phosphorylated. In summary, parasitic bacterial lipid As **12**–**18** have the following common structural features: compared to the canonical *E. coli* lipid A (**1**), the fatty chains are longer and more diverse, and only 1-position is phosphorylated.

We have accomplished the systematic chemical synthesis of these parasitic bacterial partial structures **12**–**18** and evaluated their immunostimulatory functions in human peripheral whole blood. In addition, the antagonistic effects on TLR4/MD-2 were evaluated in competitive assays against *E. coli* LPS. Parasitic bacterial lipid As **12a**, **13a**, **15**, and **16**, with three to four fatty acid chains and one normal phosphate group, showed antagonistic effects against the induction of proinflammatory cytokines, such as IL-6 and TNF-α. On the other hand, *H. pylori* lipid A **12b**, **13b** with three to four fatty acid chains and one ethanolamine phosphate group, and *P. gingivalis* lipid A **17** with five fatty acid chains and one normal phosphate group, showed agonistic IL-6 and TNF-α inducing activity, but their immunostimulatory activity was significantly lower than that of *E. coli* LPS. As mentioned before, canonical *E. coli* lipid A (**1**) is connected to the polysaccharide moiety via the bacteria-specific acidic sugar Kdo, and the immunostimulatory effect of *E. coli* lipid A (**1**) was enhanced by the introduction of Kdo [31]. In contrast, for *H. pylori* lipid A, **14a**, which is **12a** (antagonist) plus Kdo, showed more potent antagonistic effects than **12a**, and **14b**, which is **12b** (agonist) plus Kdo, switched to an antagonist [4]. In other words, in the case of *H. pylori* LPS, Kdo-lipid A, but not lipid A, is the minimum active unit. All parasitic bacterial lipid A **12**-**18** induced IL-12 and -18, which are associated with chronic inflammation, among which **12a**, **13a**, **14**–**16** selectively induced IL-12 and IL-18. Since the combination of IL-12 and IL-18 induces IFN-γ, which is involved in antitumor and anti-allergic responses, *H. pylori* lipid As, which selectively induce IL-12 and IL-18, are promising adjuvant candidates. As for LPS-mediated IL-18 induction, both the TRIF-dependent pathway [41] and TRIF-independent pathway [42] have been reported, both of which eventually lead to IL-18 induction by activating pro-IL-18 by caspases-1. In 2014, caspase-4, 5 (human), and 11 (mouse) were reported to function as cytoplasmic LPS receptors [43]. Although the molecular basis for the selective cytokine induction by parasitic bacterial lipid As is still unclear, there is a possible TLR4-independent pathway through caspase-4, 5, or 11 for caspase-1 activation upstream of IL-18 induction.

### 4.2. Symbiotic Bacterial Lipid A

Research on parasitic bacterial lipid A revealed that parasitic bacterial LPS/lipid A exhibits antagonism that favors infection in the host or weak agonistic effects that lead to chronic inflammation, suggesting that parasitic bacteria evolved to avoid the host’s innate immune response. Furthermore, it implied that parasitic bacteria could cause chronic inflammatory diseases while avoiding the bactericidal effects of acute inflammation (host immune response), suggesting that the lipid A activity deeply reflects bacterial characteristics, that is, there is a lipid A-mediated bacterial–host chemical ecology. Thus, we hypothesized that symbiotic bacteria would have immunomodulators with extremely low toxicity and that their components might be associated with maintaining homeostasis. Thus, we selected symbiotic bacterial lipid As as a safe immunomodulator pool.

Recent studies have found that LPS and lipid A from specific gut microbiota are involved in immune homeostasis and autoimmunity [44,45,46,47,48,49]. Most of these studies have focused on bacteria that inhabit the surface of the intestinal tract, such as *Bacteroides vulgatus*. Recently, the chemical and immunological properties of *B. vulgatus* LPS have been determined. The synthesis of O-antigens and examination of their affinity for defined lectins provided further insight into their beneficial role in the gut [50,51].

In our research, we focused on bacteria that colonize the gut-associated lymphoid tissues (GALT). Peyer’s plate (PP) is the major GALT located just below the intestinal epithelium throughout the entire small intestine [52,53]. PPs are also known to be the predominant sites for the initiation and regulation of endogenous IgA responses through cytokine-mediated crosstalk and cell–cell interactions consisting of dendritic cells, T cells, and B cells [54,55]. It is widely accepted that microbial stimulation is necessary for the development and maintenance of intestinal IgA production. Kiyono and Kunisawa proposed that such stimulations in PP are represented by *Alcaligenes faecalis*, which are unique Gram-negative bacteria inhabiting GALT and are responsible for the regulation of dendritic cells (DCs) to efficiently produce intestinal IgA [52,56,57]. 

*A. faecalis* is known to be an opportunistic bacterium. However, recently we focused on its LPS and found that *A. faecalis* establishes and maintains a homeostatic environment in PPs by activating the immune system by LPS without causing any harmful responses. That is, the extracted LPS fraction from *A. faecalis* showed weaker TLR4 agonistic activity than canonical *E. coli* LPS, and it could promote IL-6 induction from DCs, which, in turn, enhanced IgA production without toxicity [58]. Thus, we hypothesized that *A. faecalis* LPS tends to increase the persistence of bacteria in PPs by promoting homeostasis rather than inflammation. *A. faecalis* LPS would participate in the vigilance of the host’s immune system through the production of IgA, which in turn may promote the survival of symbiotic bacteria in PPs [58]. The extracted *A. faecalis* LPS fraction has a lower potency to induce inflammation, nitric oxide, and apoptosis than *E. coli* LPS, suggesting that these functions would also contribute to establishing symbiotic relationships with the host [7,59].

Since *A. faecalis* LPS was able to enhance IgA antibody production without toxicity, and the potency of the effect was comparable to that of *E. coli* LPS, the *A. faecalis* component was expected to be a promising candidate as a safe vaccine adjuvant. Furthermore, the antibody production enhancement of the extracted *A. faecalis* LPS was TLR4-dependent, and it was suggested that lipid A is an adjuvant function principle [58]. These TLR4-mediated immunomodulatory effects of *A. faecalis* LPS would be attributed to the chemical structure of its lipid A. Therefore, we determined the chemical structure of *A. faecalis* LPS and synthesized its lipid A to investigate the molecular basis of its immune functions.

**Figure 8:**
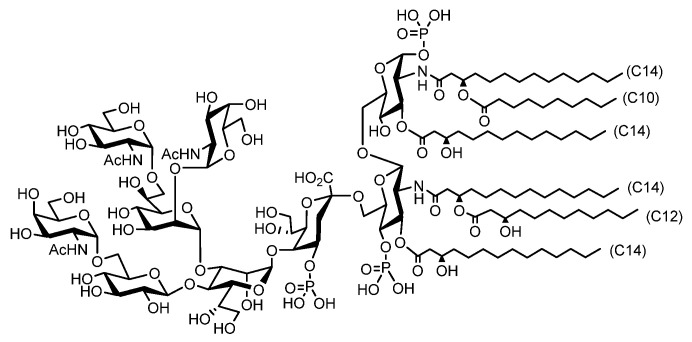
Chemical structures of *A. faecalis* LOS structures.

Canonical *E. coli* produces LPSs consisting of tens to hundreds of saccharide moieties, but some bacterial species produce lipooligosaccharides (LOS) with relatively short saccharide chains. We have identified that *A. faecalis* produces an LOS composed of nonasaccharide (Figure 8) [7].

**Figure 9:**
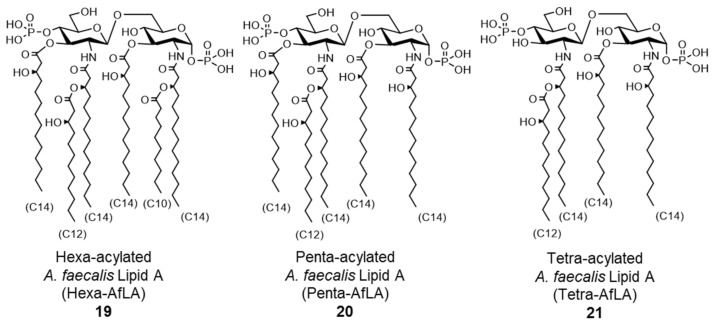
Chemical structures of synthesized *A. faecalis* lipid As.

Further structural analysis revealed that *A. faecalis* lipid A is a mixture that contains **19**–**21**, each with a different acyl chain pattern (Figure 9). Lipid A **19**–**21** were thus chemically synthesized, and their functions were evaluated using human monocytic cells. Penta-acylated *A. faecalis* lipid A **20** (penta-AfLA) and tetra-acylated *A. faecalis* lipid A **21** (tetra-AfLA) showed no immunostimulatory effect. Only hexa-acylated *A. faecalis* lipid A **19** (hexa-AfLA) showed a TLR4-dependent immunostimulatory effect comparable to extracted *A. faecalis* LOS, indicating that hexa-AfLA **19** is the active principle of *A. faecalis* LOS [7,60]. An in vivo assay with mice confirmed that hexa-AfLA **19** exhibited a similar beneficial adjuvant effect (enhancement of antigen-specific IgA and IgG production) as *A. faecalis* LOS, without toxicity [61,62]. As a safe nasal vaccine adjuvant, the efficacy of hexa-AfLA **19** has been demonstrated in *Streptococcus pneumoniae* infection models [61], indicating that it is an extremely promising adjuvant for vaccines against infectious diseases.

Since hexa-AfLA **19** from GALT resident *A. faecalis* can regulate IgA induction, which is responsible for the homeostasis of mucosal immunity, it was suggested that hexa-AfLA **19** is a critical factor in intestinal mucosal immunity. Based on bacterial–host chemical ecology research, we have focused on symbiotic bacteria inhabiting GALT, immunoregulatory tissues in the gut, and succeeded in identifying critical compounds for mucosal immunity in the intestine, and developed a promising adjuvant that can safely regulate mucosal immunity [7,60,61,62].

## 5. Lipid A in the Environment and Fermented Foods, Their Potential as Adjuvants

We selected symbiotic bacterial components as a source of safe immunoregulators and found useful adjuvant candidates, hexa-AfLA **19**. On the other hand, LPS and lipid A in environmental and fermented foods have been reported to have mild immunomodulatory functions and are attracting interest as candidates for next-generation safe adjuvants.

**Figure 10:**
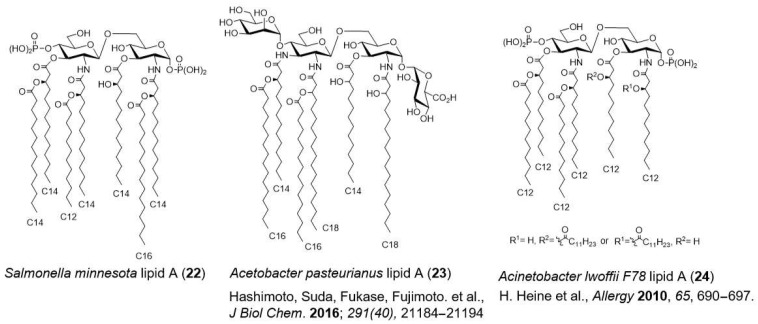
Chemical structures of lipid As in the environment and in fermented foods.

*Pantoea agglomerans*, Gram-negative bacterium widely found in soil and plants such as wheat, rice, sweet potatoes, apples, and pears, was detected in the fermentation process of rye bread [63]. *P. agglomerans* LPS showed immunostimulatory effects upon oral administration [64,65]. *P. agglomerans* lipid A is a mixture of *E. coli* lipid A (**1**) (Figure 2) and *S. minnesota* lipid A (**22**) [66] (Figure 10), both of which are agonists. Kurozu (fermented black vinegar), an Asian fermented food, contains LPS derived from Gram-negative bacteria *Acetobacter* spp., which is used in acetic acid fermentation. Recently, the chemical structures of *A. pasteurianus* LPS [67] and its lipid A **23** [68] (Figure 10) have been identified. *A. pasteurianus* LPS showed weaker immunostimulatory effects than toxic *E. coli* LPS. These LPS and lipid A from fermented foods are expected to be safe adjuvant candidates because their safety is ensured through long experience being eaten.

The hygiene hypothesis states that exposure to microorganisms in the environment during early childhood decreases the risk of developing allergic diseases. It has been suggested that LPS in the environment affects immunity development in early childhood. In the search for bacteria with an allergy-suppressing function, *Acinetobacter lwoffii* F78 was found in livestock feed [69]. *A. lwoffii* LPS selectively induces T helper 1 (Th1) cell-derived cytokines, such as IL-12 and IFN-γ, which exhibit anti-allergic effects. *A. lwoffii* F78 LPS and its lipid A **24** (Figure 10) have potential as novel adjuvants.

## 6. Self-Adjuvating Vaccines Based on Lipid A

**Figure 11:**
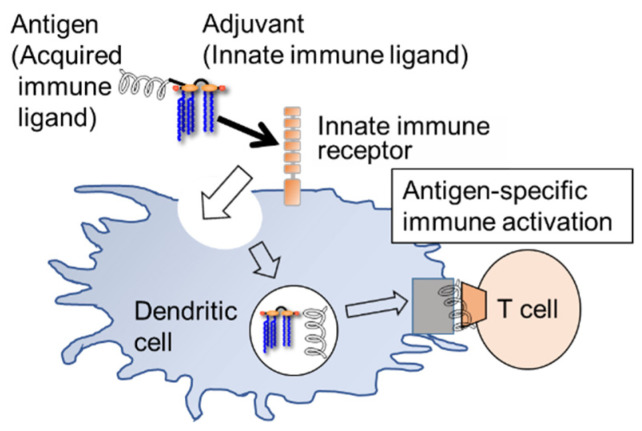
Self-adjuvanting strategy.

Finally, a strategy to enhance the adjuvant function of innate immune ligands was introduced. The self-adjuvanting strategy facilitates more efficient antibody production by complexing the antigen with the adjuvant. Recently, a significant number of studies related to this strategy, especially using lipopeptide adjuvants (Pam3CSK4, TLR2 ligand), have been reported [70,71,72,73,74,75,76,77,78]. Antigen–adjuvant complexes are actively taken up by dendritic cells via innate immune ligands (adjuvants), which activate the immune system and induce cytokine production, resulting in efficient and specific antibody production (Figure 11). The advantages of this strategy are that the antigen and adjuvant are taken up by the same dendritic cells and can trigger a specific immune response; self-adjuvating vaccines supplied by chemical synthesis are superior in quality control and safety management, as high-purity products are relatively easy to obtain. There are two main approaches to complexing antigens and adjuvants: one is based on covalent bond formation [69,70,71,72,73,74,75,76,77], and the other is based on liposomes or self-aggregate formation [79,80,81]. Here, we introduce a covalent bond formation type self-adjuvanting vaccine based on lipid A.

**Figure 12:**
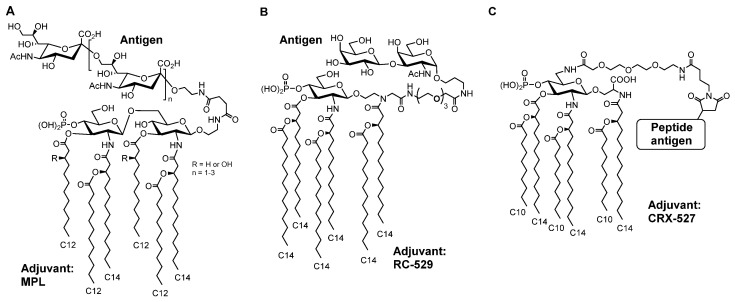
Chemical structures of adjuvant-antigen complexes; (**A**) meningococcal antigen conjugated with MPL adjuvant, (**B**) Thomsen-Friedenreich antigen conjugated with RC-529 (**11**) adjuvant, (**C**) peptide antigen conjugated with CRX-527 adjuvant.

Guo et al. synthesized MPL-based self-adjuvanting vaccines, in which the antigen was covalently bound to an MPL adjuvant. They reported a complex MPL with ganglioside GM3 [82] or α-2,9-oligosialic acid (meningococcal antigen) [83] (Figure 12A), and confirmed the enhancement of antibody production. Jiang et al. synthesized a complex of RC-529 (**11**), which mimics MPL, and Thomsen-Friedenreich antigen (a tumor-associated carbohydrate antigen) (Figure 12B) [84]. Codee et al. synthesized a complex of CRX-527, which mimics lipid A, and peptide antigen (Figure 12C), and T-cell immune response against the antigen and the specific killing of target cells expressing the antigen were observed [85].

Trumenba, a vaccine against *Neisseria meningitidis* group B, a recombinant lipoprotein with TLR2-stimulating activity, is also a type of self-adjuvanting vaccine. Furthermore, in most live-attenuated or inactivated vaccines, vaccines composed of attenuated or inactivated pathogens contain natural bacterial components, some of which would act as natural adjuvants. LPS is considered the leading natural adjuvant in vaccines derived from Gram-negative bacteria, and these vaccines can also be understood as a type of self-adjuvating vaccine.

Lipid A activity is significantly affected by subtle differences in chemical structures, such as the balance between the hydrophobic region formed by fatty acids and the hydrophilic region formed by sugar moieties and phosphate groups’ number and position and the addition of Kdo. Therefore, structural modifications often inactivate lipid A, and it is not easy to synthesize and modify the lipid A structure while maintaining its immune function. This difficulty probably leads to fewer lipid A-based self-adjuvanted vaccines than lipopeptide-based ones. However, since the lipid A derivative 3D-MPL (**10**) is the dominant adjuvant in practical use, self-adjuvanted vaccines based on lipid A seem to be very promising.

## 7. Conclusions

In this review, the structure–activity relationship of TLR4 ligands, especially lipid As, has been described. As shown in MPL and parasitic bacterial lipid A research, the structural modifications of lipid A can modulate the intensity or selectivity of TLR4/MD2 receptor activation. Therefore, cellular, humoral, and mucosal immune responses can be controlled using a specific lipid A derivative, and various lipid A derivatives, mainly MPLs, are being actively developed as adjuvants. Since the development of the clinical use of lipid A adjuvants, mainly the AS0X series (GSK), is already expanding as a component of various vaccines, such as anti-cancer, anti-protozoan, and anti-malarial vaccines, the importance of lipid A adjuvants is expected to increase further in the future.

To efficiently develop safe lipid A adjuvants, we have recently proposed a novel lipid A adjuvant development strategy based on bacterial–host chemical ecology that treats symbiotic bacterial components as a pool of safe immune modulators. The chemical synthesis and structure-related immunological function analysis of symbiotic bacterial lipid As demonstrated that hexa-acylated *A. faecalis* lipid A **19** (hexa-AfLA) is a promising adjuvant candidate with intrinsic fascinating features that cannot be derived from the detoxification of useful but toxic immunomodulators but rather on bacterial–host chemical ecology research.

The development of self-adjuvanting strategies that can further enhance the adjuvant function of lipid A by complexing antigens with adjuvants is also expected. Although lipid A is relatively difficult to modify while retaining its activity, when a simple and universal method of lipid A modification is developed to maintain its immune function, it will be a breakthrough in developing innovative self-adjuvanting vaccines.

## Data Availability

The data presented in this study are available on request from the corresponding author.

## Sample Availability

Samples of the compounds are not available from the authors.
